# The Effect of Glass Fiber Storage Time on the Mechanical Response of Polymer Composite

**DOI:** 10.3390/polym14214633

**Published:** 2022-10-31

**Authors:** Michal Jurko, Lenka Souckova, Jan Prokes, Vladimir Cech

**Affiliations:** 1 Institute of Materials Chemistry, Faculty of Chemistry, Brno University of Technology, Purkynova 464/118, CZ-612 00 Brno, Czech Republic; 2 PREFA KOMPOZITY, a.s., Kulkova 10/4231, CZ-615 00 Brno, Czech Republic

**Keywords:** glass fibers, polymer-matrix composites (PMCs), mechanical properties, environmental degradation

## Abstract

The growing demand for polymer composites and their widespread use is inevitably accompanied by the need to know their degradation behavior over a sufficiently long period of time. This study focuses on commercial glass fiber rovings, which were stored in the indoor environment for up to 11 years. Fibers with different storage times, from fresh up to the oldest, were used to produce unidirectional fiber-reinforced polyester composites that were characterized to determine their shear and flexural properties dependent on fiber storage time. A significant decrease in shear strength was observed throughout the aging of the fibers, down to a decrease of 33% for the oldest fibers. An important finding, however, was that the significant decrease in shear strength was only partially reflected in the flexural strength, which corresponded to a decrease of 18% for the oldest fibers at consistent flexural modulus.

## 1. Introduction

Glass fibers (GF) are among the primary reinforcements, accounting for almost 90% of the reinforcements used in the worldwide consumption of polymer composites. Approximately 70% of these reinforcements are embedded in a thermoset matrix. Fiberglass composite materials are used in an ever-widening range of applications, especially in the automotive and transportation sectors, the electrical/electronic industry, and the construction industry. Other markets for composite materials include pipes and tanks, agricultural equipment, wind turbine blades and the sports, consumer and marine sectors. The composite market is expected to grow by 3.8% of the compound annual growth rate (CAGR) by 2025 [1].

The most critical component of GF production is sizing, which means a thin coating applied on the GF surface, that is responsible for the processability and performance of the fibers. In addition to the film former, lubricant, and additives, the most important part of sizing is the coupling agent, which ensures adhesion between the glass surface and the resin and protects the fiber against degradation by the environment [2,3]. It was found that the strength of sized GFs is 40–80% higher than unsized (water sized) fibers due to surface protection, rather than due to the healing of flaws on a bare glass surface with a silane coupling agent [4].

The performance of fiberglass composites in indoor and outdoor environments is not constant over time and gradually decreases depending on environmental conditions. Degradation of their properties significantly shortens their service life. Degradation of the composite was therefore monitored in an outdoor environment with fluctuations in temperature and relative humidity (RH) (12–40 °C, 30–100% RH, up to 90 days [5,6]). Elevated temperature is often used in the laboratory to speed up tests in air (250 °C, 180 days [7]), water (ambient temperature, 180 days [8] or 50 °C, 100 days [9]), salt water or seawater (30–60 °C, up to 810 days [10,11,12,13]), and alkaline and acidic solutions (room temperature, 5 and 50 days [14]). The above research found that the water absorption of fiberglass composites increased with increasing temperature for environments with moisture, water, and seawater. Hygrothermal aging led to a reduction in tensile strength, shear strength, and flexural strength of composites and their modulus. This was due to the degradation of the composite constituents, fibers and the polymer matrix, as well as the reduction in adhesion at the fiber/polymer interface.

There is much less information on the aging of the GFs themselves in the indoor and outdoor environments used subsequently to make the polymer composite. Only short-term tests with elevated temperature (23–600 °C, 25 min [15]) and GF corrosion in water (25 °C, 800 h [16]) and salt water (25 and 45 °C, 4 weeks [17]) were observed. GFs with different sizing were exposed to 10%, 40%, and 80% RH for 24 h at ambient temperature without reducing their tensile strength [18]. The study by Plonka et al. [19] showed that the type of sizing coated in the laboratory affects the adhesion at the GF/polymer interface of GFs aged in an air-conditioning chamber (70 °C, 65% RH, 30 days; ambient temperature, 97% RH, 6 months). Only Peters [20] investigated the stability of commercial GFs (direct roving, 3B Fibreglass) with various sizings in the outdoor environment (8–42 °C, 40–80% RH, 4 months), which affected the shear strength of the epoxy composite. This research was supplemented by laboratory aging (50 °C, 2–3% RH, 2.5 months; 30 °C, 80% RH, 2.5 months, and their combination). The results showed a reduction of the interfacial properties of the composite in a wide range depending on the type of sizing and storage conditions. The interfacial adhesion between the fiber and the polymer matrix is the key factor affecting the performance of the polymer composite in terms of its shear and flexural properties [21].

Researchers and industrial production need to know how long stored GFs can still be used to make the polymer composite, even taking into account the possible unnecessary industrial waste. This study is therefore focused on commercial GFs that have long been stored in the indoor environment. The aged fibers were then used to form GF/polyester composites, which were tested to obtain their mechanical response in terms of shear strength and flexural strength as a function of GF storage time.

## 2. Materials and Methods

Boron-free E-glass (Advantex) fibers in the form of single-end rovings (R25H) were supplied by Owens Corning. The R25H roving had a nominal tex of 2400 g/km, a single fiber diameter of 24 µm, and a sizing designed for excellent adhesion to polyester, vinyl ester, and epoxy resins. The roving is intended for filament winding and pultrusion. Advantex glass was developed to significantly improve corrosion resistance in a wide range of aggressive environments. This glass can therefore be used for water distribution and in the market for chemical and waste piping [22]. The oldest and fresh R25H rovings with the same material characteristics were supplied by 3B Fibreglass. The production date of the rovings ranged from February 2010 to June 2021. The sizing formulation of the R25H rovings used by the manufacturer may have changed over the years, although the roving specification was still the same. Owens Corning’s product information published in 2012 and 2020 attests to the fact that there has been no change in the technical characteristics (mechanical properties) of R25H roving and polymer composites made from it. We therefore assume that the achieved results well represent the properties of the stored fibers and can be generally applicable. Owens Corning recommends storing glass fiber products using the original packaging material (shrinkable foil) in a cool, dry place until use. Shelf life is not known for proper storage, but to ensure optimal performance, the manufacturer recommends re-testing after three years from the date of original manufacture [22]. 3B Fibreglass considers ideal storage conditions to be between 15 °C and 35 °C and relative humidity between 35% and 75% [23].

All rovings were stored in open original packaging in the production hall, where the temperature ranged from 10 °C to 35 °C and the relative humidity from 30% to 95%. Prior to the production of composite samples, the roving was stored under air-conditioned laboratory conditions (20–22 °C, 40–45% RH) for at least one week.

Each batch of stored GFs was used to manufacture rectangular composite beams using the hand laying method under laboratory conditions. To prevent the presence of dust particles on the surface of stored fibers, several hundred meters of roving were removed from the bobbin prior to sample production. GFs were embedded into unsaturated polyester resin (isophthalic) Distitron 183 B1 (Polynt S.p.A., Italy) to form a GF/polyester composite in a silicon rubber mold with a size of 3 × 10 × 200 mm^3^ or 3 × 10 × 330 mm^3^ for the short-beam shear test or the flexural test, respectively. The chosen height and width of the molds comply with standards. A total of 13 fiber bundles were gradually inserted in parallel into the mold and impregnated with the resin, which was finally cured at 140 °C for 1 h. This long composite beam with a fiber volume fracture of 39% was cut into specimens, which were ground to a given size on a metallographic grinder according to the type of test, and these specimens were stored in a desiccator before further testing. The void content in the samples is approximately 2 vol%.

Short-beam composites measuring 3 × 10 × 18 mm^3^ were tested in a three-point bending according to the standard ASTM D2344 [24]. The ratio of span length to specimen thickness was 4. Six specimens were tested for each storage time. In the short-beam shear test, the short-beam strength is related to the maximum applied load, *P_max_*, the specimen width, *b*, and the thickness, *h*, according to this relation

(1) $$\tau_{max}=\frac{3P_{max}}{4bh}\;.$$

The test speed was set at a crosshead movement of 1 mm/min. Load-displacement curves were monitored using a Materials Testing Machine (AllroundLine Z010 TE, ZwickRoell, Germany). Delaminated short-beams after fracture were sputtered with gold to improve their surface conductivity, and then observed using a scanning electron microscope (SEM, JSM-7600F, Jeol, Japan).

To determine the flexural properties, three-point bending tests were carried out with longer composite beams measuring 3 × 10 × 90 mm^3^ according to ASTM D790 [25]. Six samples were tested for each storage period, approximately 18 months after the shear test. Load-deflection curves were used to determine the flexural strength, flexural modulus, and elongation at break. The support span, *L*, was 60 mm. The support span-to-depth ratio was thus 20 to 1, and since the deflection exceeds 10% of the support span, the flexural strength corresponding to the maximum stress on the outer surface of the specimen is approximated by equation [25]

(2) $$\sigma_{max}=\frac{3P_{max}L}{2bh^{2}}\;\left[1+6{\left(\frac{D_{max}}{L}\right)}^{2}-4\frac{hD_{max}}{L^{2}}\right]\;,$$

where *D_max_* is the deflection of the centerline of the specimen at the middle of the support span corresponding to the maximum applied load, *P_max_*. The crosshead speed was 2 mm/min for the flexural test.

## 3. Results and Discussion

### 3.1. Shear Properties

**Figure 1:**
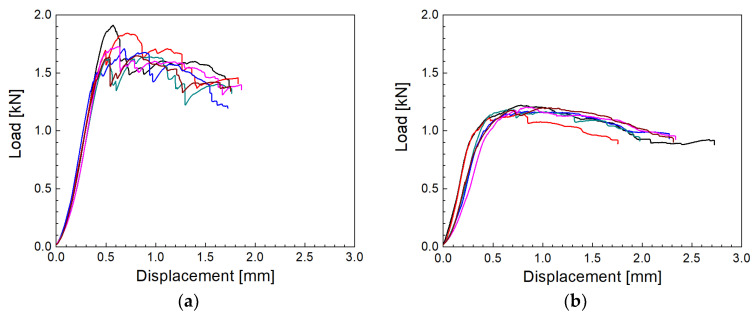
Load-displacements curves as a result of a short-beam shear test for (**a**) fresh and (**b**) the oldest fibers.

**Figure 2:**
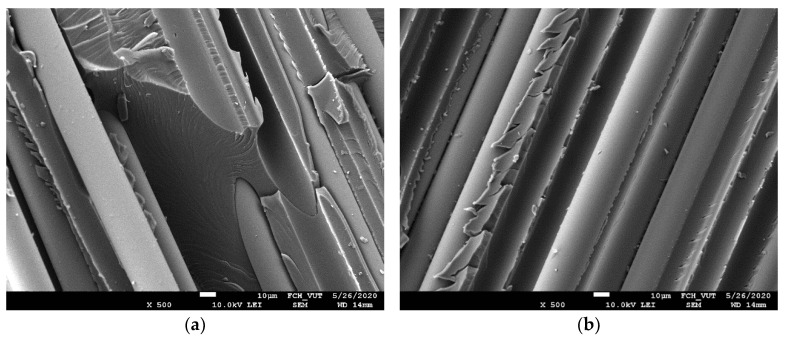
SEM micrograph of fractured composite beam reinforced with (**a**) fresh fibers and (**b**) glass fibers that were stored for 116 months.

Short composite beams reinforced with fresh up to the oldest GFs were tested by three-point bending. The shear stress induced in a short-beam subjected to the bending load is directly proportional to the magnitude of the applied load, and independent of the span length. While bending stresses are directly proportional to both the applied load and the span length. The support span of the short-beam is thus kept short, so that an interlaminar shear failure occurs before the bending failure [26]. Mid-plane interlaminar failure was identified for each sample tested. The load-displacements curves corresponding to the fresh and oldest GFs are shown in Figure 1a,b, respectively. The maximum of the curves is more pronounced and at higher loads for fresh fibers than in the case of the smooth round shape belonging to the oldest fibers. The character of the curves and their maximum load indicate a significantly higher interfacial adhesion of fresh fibers. The SEM micrographs in Figure 2 show the fracture surface of a short composite beam reinforced with fresh (Figure 2a) and oldest (Figure 2b) GFs after its interlaminar failure. The fibers are partially covered with a polyester resin with hackles typical of the interlaminar shear failure mode, which indicates high interfacial adhesion. Micrographs of the same characteristics were observed for GFs of all storage times.

**Figure 3:**
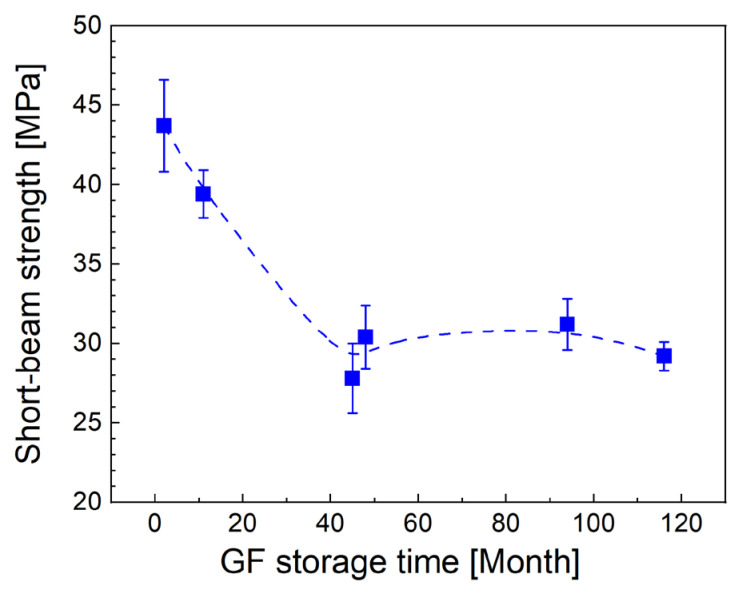
Short-beam strength for GF/polyester composites reinforced with glass fibers with different storage times.

The short-beam strength as a function of fiber storage time is plotted in Figure 3. A smoothing spline was used to fit the data only to capture the dependence trend. The initial shear strength of 43.7 MPa dropped to 39.4 MPa after only 11 months of fiber storage. Another significant decrease occurred after 45 months and did not change significantly until 116 months, and in this time range the values fluctuate between 27.8 and 31.2 MPa. The silane coupling agents as part of the sizing are responsible for the formation of a siloxane network (interphase), which makes it possible to connect the GF surface to the polymer network (matrix) through strong chemical bonds. However, this siloxane bond (Si–O–Si) is hydrolytically unstable, and when GFs are stored in a humid environment, water molecules first physisorb on the surface of the sized fibers and then gradually diffuse into the sizing, debonding the siloxane network and its anchorage to the fiber surface [3,27]. The disrupted siloxane network is then unable to efficiently transfer the mechanical stress of the loaded composite from the polymer matrix to the fiber. The siloxane bonds are only partially reformed if water molecules are removed during the drying process [28]. The functional group of the coupling agent by which the siloxane network binds to a given polymer matrix can also be hydrolyzed, thereby reducing sizing reactivity [20]. Peters also points to the possibility of reduced sizing solubility when exposed to heat in a dry environment [20]. These changes in sizing during fiber storage could affect roving wettability and lead to a different void content at the fiber/polymer interface. It can therefore be expected that the reduced short-beam strength with increasing storage time (Figure 3) is then a consequence of sizing degradation. The increased temperature accelerates the diffusion of water due to the Arrhenius law and thus speeds up the degradation process [27].

### 3.2. Flexural Properties

**Figure 4:**
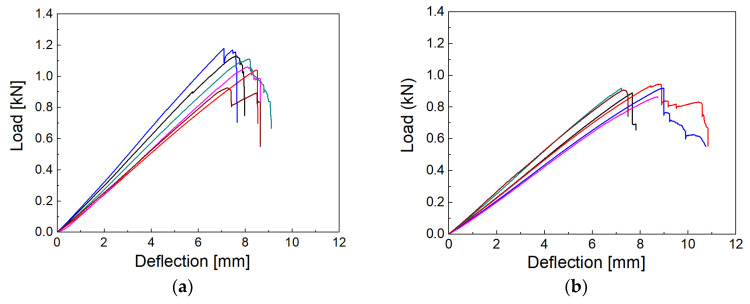
Load-deflection curves corresponding to the bending test of a composite reinforced with (**a**) fresh and (**b**) the oldest fibers.

In a three-point bending test with longer composite beams with dimensions of 3 × 10 × 90 mm^3^, the beam is in a combined stress state with maximum tension at the lower surface, maximum compression at the upper surface, and maximum interlaminar shear at the mid-plane [26]. The load-deflection curves for the fresh (Figure 4a) and the oldest (Figure 4b) GFs look similar, but the average maximum load is higher for the fresh fibers. Additionally, the decrease in load following the maximum of the curve at lower deflection is evident in fresh fibers affected by a sudden decrease in tension on the fibers due to composite failure.

**Figure 5:**
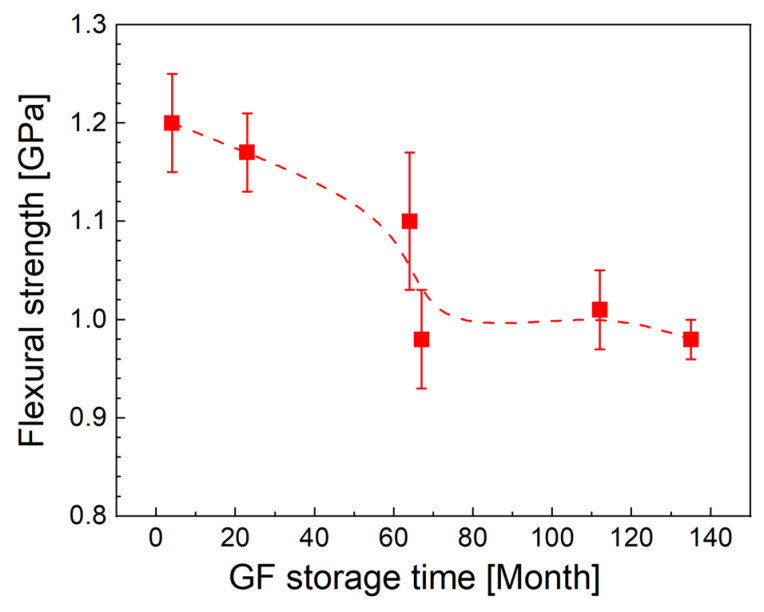
Flexural strength for GF/polyester composites reinforced with fibers with different storage times.

The flexural strength of GF/polyester composites reinforced with GFs of different storage times is shown in Figure 5. Like the short-beam strength, the flexural strength gradually decreases when fibers with longer storage period are used. The flexural strength of 1.20 GPa for fresh fibers dropped slightly to 1.17 GPa after 19 months. This is followed by a more significant decrease to 1.04 GPa corresponding to the average flexural strength for a storage period of 64 to 67 months. We expect that the sharp change in flexural strength between 64 and 67 months is merely a fluctuation in the data due to the mean value determined from the six samples. A much higher number of samples could lead to less variation in the data. A further decrease in strength with storage time is no longer noticeable and the values range between 0.98 and 1.01 GPa. The flexural modulus and the elongation at break were around the mean values of (23.9 ± 2.3) GPa and 0.042 ± 0.005, respectively, regardless of the age of the fibers. A close look at the individual load-deflection curves shows that the reduced flexural strength coincidentally corresponds to a lower flexural modulus, resulting in an approximately consistent value of elongation at break.

### 3.3. Shear versus Flexural Properties

**Figure 6:**
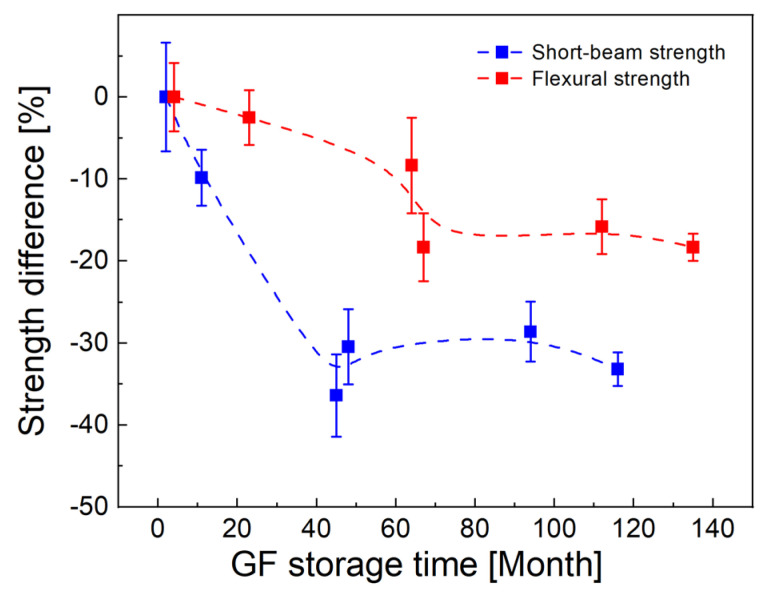
Effect of fiber storage time on short-beam strength and flexural strength of GF/polyester composites.

The time-dependent difference in the short-beam strength and the flexural strength with respect to their initial values for fresh fibers is shown in Figure 6. A significant decrease in shear strength of 10% after 11 months has a response in only a 3% decrease in flexural strength. After 45 months, the short-beam strength is already approximately 32% lower and does not change significantly with increasing storage time. This significant decrease in shear strength has the effect of reducing the flexural strength, which is around 85% of the initial value. The specific value of flexural strength is 18% lower for 135-month-old fibers. A correlation can be expected between the flexural strength and the short-beam strength because the shear properties affect the mechanical response of the polymer composite in the bending test [29]. The time-dependent degradation of sizing is therefore partly reflected in the decrease in flexural strength (Figure 6). However, the flexural strength of the composite can also be affected by GF degradation (corrosion), which reduces the tensile strength of individual fibers. The results of GF short-term aging under ambient conditions do not indicate a reduction in the GF tensile strength [15,16,17,18,30], but the effect of long-term aging on fiber strength is unknown. The fact that there was no decrease in the tensile strength of the fibers over time is also indicated by approximately the same flexural modulus of the composite beams.

## 4. Conclusions

Commercial rovings were stored in the indoor environment (10–35 °C, 30–95% RH) for zero to eleven years. The storage conditions were similar to those required by the manufacturer, only the relative humidity was up to 95% compared to the required maximum value of 75% and the minimum temperature was 10 °C compared to the required 15 °C, and the rovings were stored in the open original packaging. The GF rovings stored for different periods of time were then used to make the GF/polyester composite that was tested by short-beam shear and bending tests to characterize its shear and flexural properties depending on GF storage time. The short-beam strength decreased from 43.7 MPa to 29.2 MPa within 114 months, indicating a significant reduction in the interfacial properties of the composite depending on the GF storage time. When a significant decrease occurred after 45 months of storage, and the shear strength did not change significantly thereafter. The deterioration of the interfacial adhesion between the composite constituents inevitably resulted in a decrease in the flexural strength from 1.20 GPa to 0.98 GPa for composites with a fiber volume fracture of 39%. A relative comparison of the changes in both strengths points to the important finding that a significant decrease in shear strength is only to a limited extent reflected in a decrease in flexural strength. Thus, a 10% decrease in shear strength during the first two years was reflected in only a 3% decrease in flexural strength. Similarly, a 33% decrease in shear strength after ten years manifested itself as an 18% decrease in flexural strength. The flexural modulus and elongation at break appeared to be consistent over time. A more detailed time study with a shorter interval (several months) of fiber storage time would specify the time course of the shear and flexural properties of the composite.

## Data Availability

The data that support the findings of this study are available from the corresponding author upon reasonable request.

## References

[1] Composites Manufacturing, 2020 State of the Industry Report. http://compositesmanufacturingmagazine.com/2020/01/2020-state-of-the-industry-report/2/.

[2] Dwight D.W., Kelly A., Zweben C. (2000). Glass fiber reinforcements. Comprehensive Composite Materials.

[3] Thomason J.L. (2019). Glass fibre sizing: A review. Compos. Part A.

[4] Yang L., Thomason J.L. (2013). Effect of silane coupling agent on mechanical performance of glass fibre. J. Mater. Sci..

[5] Swain S., Beura S., Thatoi D.N., Chakraverty A.P., Mohanty U.K. (2019). Durability of GFRP composite exposed to outdoors weathering. Compos. Commun..

[6] Beura S., Sahoo S.R., Thatoi D.N., Mohanty U.K., Chakraverty A.P. (2021). Stability of GFRP composites with varied fractions of reinforcement exposed to ageing processes outdoors. Polym. Polym. Compos..

[7] Li J., Fan W., Liu T., Xue L., Yuan L., Dang W., Meng J. (2022). Hardness and flexural performance of 3D orthogonal carbon/glass fibers hybrid composites under thermal-oxidative aging. J. Ind. Text..

[8] Suhas K., Gowrishankar M.C., Manjunath S., Ritesh B., Gurumurthy B.M. (2021). Durability prediction analysis on mechanical properties of GFRP upon immersion in water at ambient temperature. Cogent. Eng..

[9] Quino G., Tagarielli V.L., Petrinic N. (2020). Effects of water absorption on the mechanical properties of GFRPs. Comp. Sci. Technol..

[10] Gellert E.P., Turley D.M. (1999). Seawater immersion ageing of glass-fibre reinforced polymer laminates for marine applications. Compos. Part A.

[11] Hu Y., Lang A.W., Li X., Nutt S.R. (2014). Hygrothermal aging effects on fatigue of glass fiber/polydicyclopentadiene composites. Polym. Degrad. Stabil..

[12] Yang S., Liu W., Fang Y., Huo R. (2019). Influence of hygrothermal aging on the durability and interfacial performance of pultruded glass fiber reinforced polymer composites. J. Mater. Sci..

[13] Li S., Guo S., Yao Y., Jin Z., Shi C., Zhu D. (2021). The effects of aging in seawater and SWSSC and strain rate on the tensile performance of GFRP/BFRP composites: A critical review. Constr. Build. Mater..

[14] Belloul N., Hamadache H., Benyahia A.A., Serier A. (2015). Effect of the aggressive environment on the damage of a glass polyester composite developed by hand layup process. Adv. Mater. Sci. Eng..

[15] Jenkins P.G., Yang L., Liggat J.J., Thomason J.L. (2015). Investigation of the strength loss of glass fibre after thermal conditioning. J. Mater. Sci..

[16] Nagae S., Otsuka Y. (1996). Effect of sizing agent on corrosion of glass fibre reinforced plastics (GFRP) in water. J. Mater. Sci. Lett..

[17] Gu H. (2009). Tensile behaviours of quartz, aramid and glass filaments after NaCl treatment. Mater. Des..

[18] Brown E.N., Davis A.K., Jonnalagadda K.D., Sottos N.R. (2005). Effect of surface treatment on the hydrolytic stability of E-glass fiber bundle tensile strength. Comp. Sci. Technol..

[19] Plonka R., Mäder E., Gao S.L., Bellmann C., Dutschk V., Zhandarov S. (2004). Adhesion of epoxy/glass fibre composites influenced by aging effects on sizings. Compos. Part A.

[20] Peters L., Davies P., Rajapakse Y.D.S. (2018). Influence of glass fibre sizing and storage conditions on composite properties. Durability of Composites in a Marine Environment 2.

[21] Palesch E., Knob A., Plichta T., Cech V. (2018). Functional interlayers with controlled adhesion developed for polymer composites. Thin Solid Film..

[22] Owens Corning, R25H Type 30 Roving Product Data Sheet. https://dcpd6wotaa0mb.cloudfront.net/mdms/dms/CSB/10024135/10024135-R25H-Product-Data-Sheet.PDF?v=1591936368000.

[23] 3B Fibreglass, Datasheet R25H Direct Rovings. https://www.3b-fibreglass.com/sites/default/files/products-data-sheets/R25H_Direct-Roving1.pdf.

[24] (2000). Standard Test Method for Short-Beam Strength of Polymer Matrix Composite Materials and Their Laminates.

[25] (2003). Standard Test Methods for Flexural Properties of Unreinforced and Reinforced Plastics and Electrical Insulating Materials.

[26] Adams D.F., Kelly A., Zweben C. (2000). Test methods for mechanical properties. Comprehensive Composite Materials.

[27] Plueddemann E.P. (1991). Silane Coupling Agents.

[28] Ishida H., Koenig J.L. (1980). Effect of hydrolysis and drying on the siloxane bonds of a silane coupling agent deposited on E-glass fibers. J. Polym. Sci..

[29] Wu H.F., Dwight D.W., Huff N.T. (1997). Effects of silane coupling agents on the interphase and performance of glass-fiber reinforced polymer composites. Comp. Sci. Technol..

[30] Thomason J., Jenkins P., Yang L. (2016). Glass fibre strength-a review with relation to composite recycling. Fibers.

